# Modular annular photocatalytic membrane reactor for the degradation of micropollutants: Design and application

**DOI:** 10.1016/j.mex.2025.103478

**Published:** 2025-07-01

**Authors:** Michael S. Leupold, Max Reuschenbach, Gerrit Renner, Anam Asghar, Klaus Kerpen, Lukas Fischer, Torsten C. Schmidt

**Affiliations:** a Instrumental Analytical Chemistry, University of Duisburg-Essen, Universitätsstr.5, Essen 45141, Germany; b Centre for Water and Environmental Research (ZWU), University of Duisburg-Essen, Universitätsstr.2, Essen 45141, Germany; c Technical Chemistry II, University of Duisburg-Essen, Universitätsstr.5, Essen 45141, Germany; d IWW Water Center, Moritzstr.26, Mülheim an der Ruhr 45476, Germany

**Keywords:** Heterogenous photocatalysis, Modular annular photocatalytic membrane reactor (MAPMR), Amoxicillin, UV Filter, Design and application of the Modular Annular Photocatalytic Membrane Reactor (MAPMR)

## Abstract

This study presents the Modular Annular Photocatalytic Membrane Reactor (MAPMR), designed to address key challenges in conventional photocatalytic reactors, such as uniform light distribution, efficient photocatalyst recovery, and a precise control over reaction conditions. The MAPMR features:

1) A modular, annular configuration with vertically stacked photocatalyst-immobilized membranes surrounding the light source, ensuring uniform light distribution.
2) Continuous separation and recovery of photocatalysts, while providing precise control over parameters such as reaction time, temperature, and light intensity.
3) Use of UV-cut filters to block light wavelength below specific thresholds.

The reactor was tested with TiO₂-decorated polyethersulfone (PES) membranes (TiO₂-PES) and equipped with selective UV cut-off filters to selectively assess individual micropollutant degradation processes. These filters block wavelengths below 296 nm, 325 nm, and 405 nm, facilitating separate studies of UV- and visible light-driven photocatalysis while preventing direct photolysis. The reactor's modular design facilitates easy membrane replacement, supports diverse operational modes, and integrates in-line and on-line monitoring for real-time analytical insights. Amoxicillin (AMX), a model organic pollutant, was used as the probe compound to evaluate reactor performance with potassium nitrate as a UV cut-off filter (λ < 325 nm) providing precise evaluation of both photocatalytic and photolytic AMX degradation.

## Graphical abstract

**Figure:**
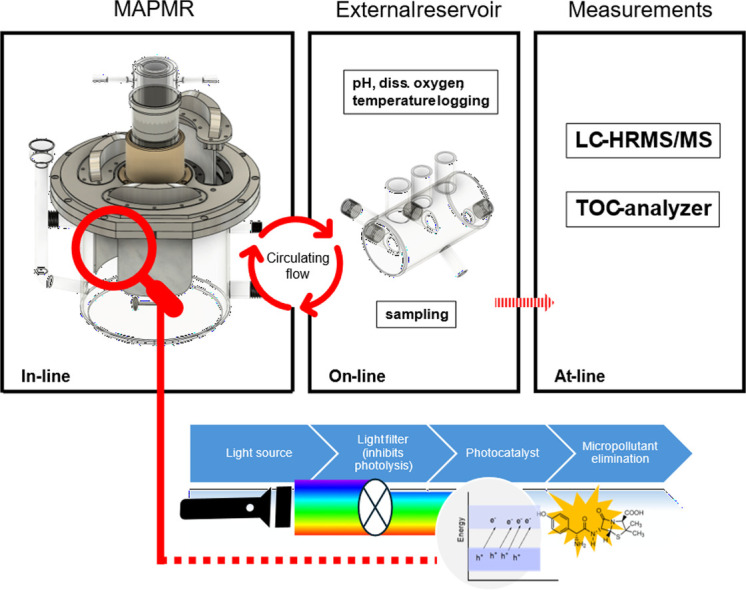

Specifications table

**Table:** 

Subject area:	Environmental Science
More specific subject area:	*Heterogenous photocatalysis on micropollutants*
Name of your method:	*Design and application of the Modular Annular Photocatalytic Membrane Reactor (MAPMR)*
Name and reference of original method:	*N/A*
Resource availability:	*Data will be made available on request.*

## Background

In recent decades, extensive research on the photo(catalytic) degradation of organic compounds for water and wastewater treatment has driven advancements in catalyst materials, reactor configurations, and operational designs, significantly enhancing treatment efficiency [1]. While substantial efforts have been made towards enhancing photocatalyst activity, comparatively less attention has been given to developing photoreactors and optimizing operational parameters.

Photocatalytic reactors are classified into slurry-type and fixed-bed configurations, each with distinct advantages and limitations [2]. With their simple design and enhanced catalyst-contaminant interactions, slurry-type reactors are widely used in water treatment. However, the low efficiency of incident light, mainly when the catalyst concentration is too high, along with photocatalyst recovery, remains a significant drawback [2]. While annular photoreactors improve light absorption by incorporating a central light source, uniform catalyst distribution, and low catalyst recovery remain challenging [3]. On the other hand, fixed-bed reactors address recovery issues by immobilizing the catalyst, but they encounter limitations, including mass transfer, non-uniform light distribution, and photo absorption [4].

Integrating photocatalysis with membrane filtration presents a promising solution for high-quality permeate production [5]. However, membrane fouling caused by the deposition of photocatalyst nanoparticles, which leads to flux decline, remains a significant challenge. Immobilizing photocatalysts directly on membranes mitigates this issue by eliminating the need for a separate separation step and enabling efficient catalyst recovery and reuse. However, this approach can reduce photocatalytic efficiency in conventional reactor configurations where membranes are stacked horizontally due to non-uniform light distribution and complicate membrane removal in continuous flow systems [6].

These ongoing challenges highlight the need to design and optimize photoreactors that effectively address key issues, including mass transfer, uniform light distribution, efficient photocatalyst recovery and reuse, and streamlined membrane handling. Our approach aims to tackle these limitations by introducing an optimized solution that improve reactor performance and enables more effective integration of photocatalysis and membrane filtration. The key features of our approach include:

• Design of **Modular Annular Photocatalytic Membrane Reactor (MAPMR)**, featuring vertically stacked, photocatalyst-immobilized membranes surrounding the light source to achieve uniform light distribution.
• Flexible replacement of membrane units enabled by a mounting bracket.
  As a proof of concept, amoxicillin (AMX), a widely prescribed antibiotic, was selected as the probe compound to evaluate photocatalytic degradation in the MAPMR, utilizing vertically stacked TiO_2_ decorated polyethersulfone (PES) membrane as the heterogeneous photocatalyst. AMX absorbs UV light at $\lambda_{\text{max}}$= <300 nm, allowing photolysis (photoinduced dissociation, PID) alone to fully degrade the compound without the need for photocatalysis. Direct and indirect photolysis in parallel to photocatalysis presents a challenge in accurately evaluating photocatalytic materials. To excite TiO_2_, emission below 400 nm is required, but suitable light sources often also emit UVB radiation, and therefore, the extent of degradation due to photocatalysis can be difficult to determine due to simultaneous photolysis. This leads to another key feature of our approach:
• Using different UV cut-off filters in the annular region around the light source blocks wavelengths below specific thresholds. This enables the isolation ('turn on') of visible light alone, followed by the selective and stepwise addition of UVA, UVB, and UVC regions.
  In the MAPMR, this feature ensures that AMX degradation occurred exclusively through photocatalysis, thereby enabling a more precise evaluation of photocatalytic performance.

## Method details

### Phase 1: design of MAPMR

**Fig. 1:**
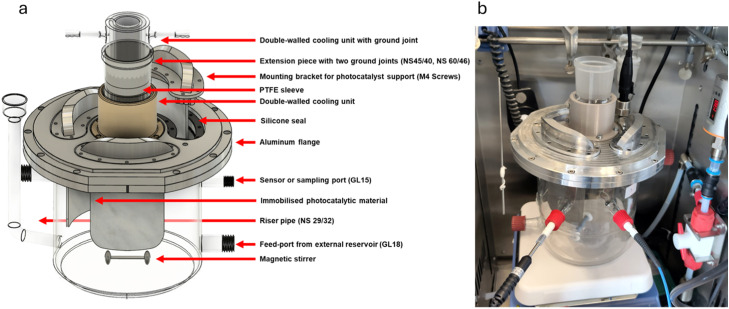
a) 3D-Model of the MAPMR created by using Autodesk Fusion 360 and b) manufactured MAPMR in use.

The 3D model of the MAPMR designed in this study is shown in Fig. 1a, with detailed engineering drawings and dimensions provided in the supplementary information (S11).

The MAPMR consists of three sections: (i) the reaction chamber, fabricated from borosilicate glass; (ii) an aluminium metal flange forming the upper reactor section, which incorporates interchangeable mounting brackets to support immobilized photocatalyst materials, providing flexibility for catalyst replacement; and (iii) the annular quartz tube, centrally positioned within the reactor, designed for the central mounting of the light source and the integration of a cooling jacket.

The reaction chamber, which constitutes the lower section of the MAPMR, features four threaded ports and a riser pipe. The lower and upper ports on opposite sides of the reactor serve as inlet and outlet connections, providing flexibility for various operational modes such as single-pass, semi-batch, or batch configurations. Additional ports are allocated for in-line monitoring, facilitating the integration of a temperature sensor and a USB spectrometer for real-time temperature and lamp spectrum measurements, respectively. Sensors are inserted through these ports using GL15 threaded caps (DIN 168), with PTFE tape used to seal the optics, ensuring waterproofing and a secure, and reliable fit that maintains proper functionality. The riser pipe, sealed with a glass stopper during photocatalytic reactions, is incorporated into the reactor design to facilitate solution flushing with inert gas or oxygen as needed, thereby providing precise control over the oxygen concentration.

The MAPMR design also features an anodized aluminium split flange, positioned at the top to establish a secure and tight connection between the glass lining of the reactor and the reactor lid. The anodization process enhances the chemical resistance of the flange, while a silicone metal-to-glass seal is used to ensure a tight connection between the flange and the glass lining, maintaining material compatibility. The upper planar surface of the reactor chamber was 3D scanned, and the resulting model was used to design and fabricate the flange with high precision. An aluminium plate, the reactor lid, is mounted on the flange, and designed to accommodate the annular quartz tube and bracket holders. This configuration creates a sealed environment, which is crucial monitorable experimental conditions. Detailed blueprints of flanges and reactor lid are provided in the SI, section S11.

**Fig. 2:**
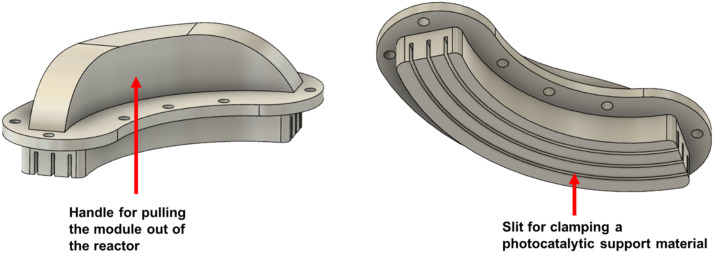
3D-model of photocatalyst mounting bracket.

Three interchangeable bracket holders (Fig. 2), vertically stacked within the reactor, are designed to support immobilized photocatalytic materials. This design offers flexibility in material selection and allows for easy replacement. The holders are curved in shape and securely affixed to the reactor lid using a metric M4 screw, ensuring a consistent distance between the immobilized catalytic surface and the light source, which promotes uniform light distribution across the entire surface. To allow flexible and precise adjustment of the material-light source distance, the inner side of each holder features slits with optional distances of 54 ± 0.5, 59 ± 0.5, and 64 ± 0.5 mm, depending on the selected slit (Fig. 2). This adaptable design can accommodate various photocatalytic materials, such as coated metal meshes, nets, membranes, sponges, or 3D-printed structures. These support materials, immobilized with a photocatalyst, can be securely clamped or glued into the bracket holder slits, as shown in Fig. 2. This setup allows the membrane to operate in a flow-by mode, ensuring that the removal of organic pollutants is not due to filtration or size exclusion, but rather results from photocatalytic degradation. This configuration also reduces membrane fouling, which could otherwise hinder photocatalytic efficiency by blocking the active sites or limit light penetration. A silicone seal between the bracket holders and the reactor lid ensures a sealed environment, enabling controlled atmospheres (e.g., inert gas or oxygen).

The MAPMR features a cylindrical quartz glass container, the annular quartz tube, specifically designed to centrally house the light source and integrate a cooling jacket (SI, S10). The annular shape facilitates uniform light distribution, with the UV light source positioned at the center of the tube. Additionally, the annular tube includes a void region between its inner and outer tubes, allowing cooling water to circulate from a water bath through two integrated ports, ensuring efficient temperature control within the reactor. This annular tube is mounted inside the reactor via a borosilicate glass cylindrical sleeve extending outward from the lid. A PTFE sleeve is clamped between them to prevent the quartz and borosilicate glass components from becoming stuck. Detailed blueprints of the components are provided in the SI (S11).

The MAPMR design supports semi-batch operation by incorporating an external borosilicate glass reservoir connected to the reactor via ports. This configuration provides flexibility for adjusting reaction conditions, facilitates sampling, and allows for the integration of additional analytical equipment (see SI, S10). The external reservoir features a round design, promoting passive mixing during operation. A one-way valve is installed in the line connecting the external reservoir to the reaction chamber to prevent backflow and overflow when the pump is turned off. The MAPMR is housed within a safety cabinet for safe and secure operation, as shown in Figure S8.

**Fig. 3:**
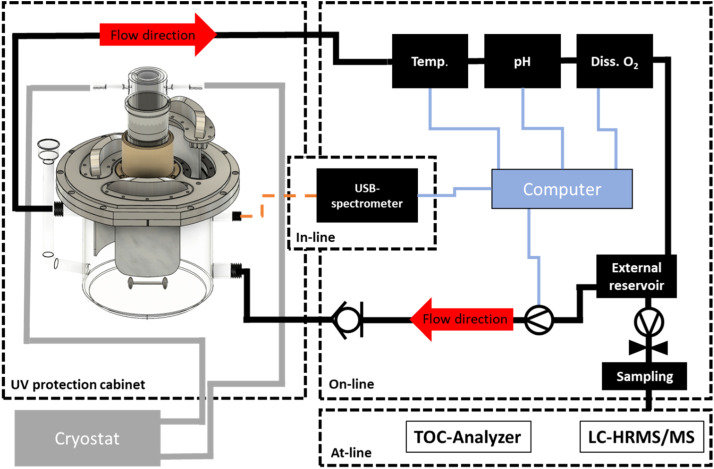
Schematic of the Semi-batch reactor setup including in-line, on-line and at-line analytics.

The MAPMR system has a total working volume of 3 L, distributed as follows: 2.25 L in the reaction chamber, 0.6 L in the external reservoir, and 0.15 L in the hoses, connectors, the one-way valve, and the pump. The pump can operate at a total flow rate of 10 L/min. A schematic of the reactor setup is shown in Fig. 3.

### In-Line and on-line monitoring

The MAPMR design incorporates in-line monitoring of process parameters, including the UV spectrum of the lamp and the reactor's internal temperature, using sensors installed via two threaded ports. Real-time measurements of pH, temperature, and dissolved oxygen are conducted on-line by positioning sensors within the external reservoir. A specialized arrangement is implemented to facilitate in-line analyte spectra measurement and qualitative evaluation of degradation performance. This involves withdrawing a small sample volume from the reservoir using a peristaltic pump and directing it through a flow-through cuvette connected to a USB spectrophotometer. The configuration is detailed in SI (section S9, Figure S7). A custom LabVIEW application continuously monitors in-line and on-line data during the experiment. The collected parameters are stored in text or CSV format linked to the corresponding experiment.

### Phase 2: synthesis of photocatalytic membrane

**Fig. 4:**
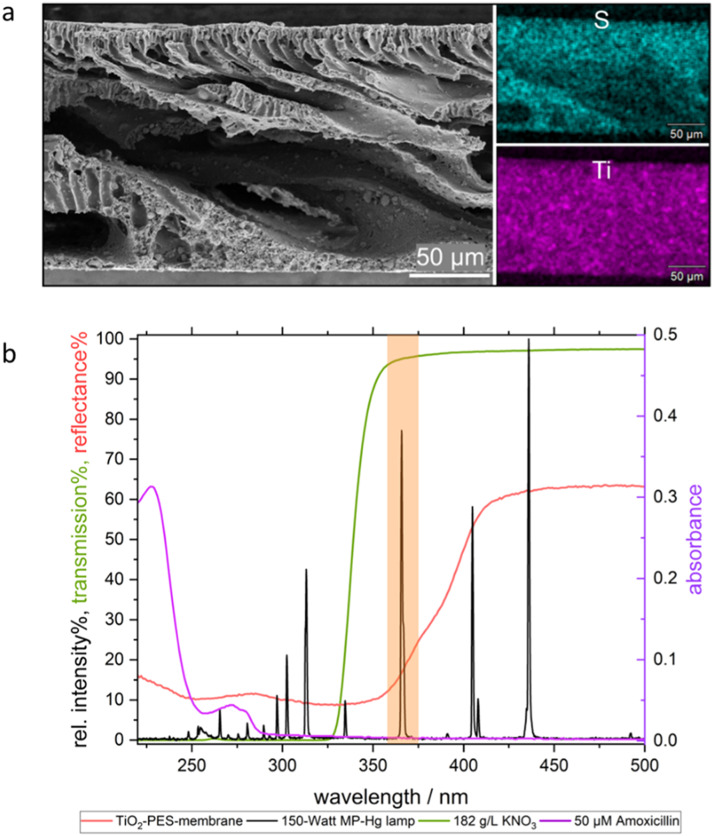
a) SEM image und EDX mapping of the cross-section of TiO_2_-PES, b) Normalized 150-Watt MP Hg Lamp spectra (black), absorption spectra of 50 µM AMX in pure water (purple), reflectance spectra of the TiO_2_-PES membrane (red), transmission spectra of 182 g/L KNO_3_ in pure water (green). Orange area indicates the lamp peak expected to excite TiO_2_-PES.

To obtain an immobilized photocatalyst for demonstrating the practical application of MAPMR, a TiO_2_-decorated porous polyethersulfone (PES) membrane (TiO_2_-PES) was synthesized following a previously described procedure [7,8]. In brief, the synthesis involved preparing a casting solution by mixing commercial P25 TiO_2_ particles (15 wt%), cationic ionomer Fumion (4 wt%, binder), and PES (20 wt%, matrix polymer) in *N*-methylpyrrolidone (NMP, solvent). The porous flat-sheet membrane was then fabricated by film casting (250 µm) of this solution, followed by immersion in a water precipitation bath. The Ti content of TiO_2_-PES was determined to be 16 wt% by quantitative EDX, with TiO_2_ uniformly distributed across the porous cross-section (Fig. 4a). The contact angle measurements are provided in the SI (Section S4). The band gap of the TiO_2_-PES was calculated using the method described in section S3 (SI) and found to be 3.12 eV, which is slightly lower than that of powdered TiO_2_ (3.20 eV, Table S1). The slight decrease in band gap from 3.20 eV to 3.12 eV suggests that the integration of TiO_2_ in the membrane matrix may be influencing its electronic properties, possibly due to surface complexation by either PES or the cationic polymer binder (Fumion).

### Phase 3: photocatalytic degradation of AMX: use of UV cut-off filter

Prior to the experiments, the UV–Vis spectrum of AMX (50 µM) and the light reflectance spectrum of the TiO_2_-PES membrane were determined as described in section S3 (SI). As shown in Fig. 4, AMX absorbs light at wavelengths below 300 nm, which overlaps with the emission spectrum of the medium-pressure lamp used in this study. This indicates that direct photolysis of AMX takes place, if no UV cut-off filter is applied. Furthermore, the reflectance spectrum of TiO_2_-PES reaches its minimum at 350 nm (Fig. 4b), suggesting that incident UV light at this wavelength provides sufficient energy to overcome the band gap of the immobilized TiO_2_ and generate electron-hole (e^-^/h^+^) pairs. Therefore, in applications where the absorption spectrum of AMX overlaps with the emission spectrum of the light source and the excitation wavelength of the photocatalyst, the system can effectively integrate photolytic and photocatalytic processes. However, the explicit contribution of the photocatalyst in such scenarios remains unclear.

**Fig. 5:**
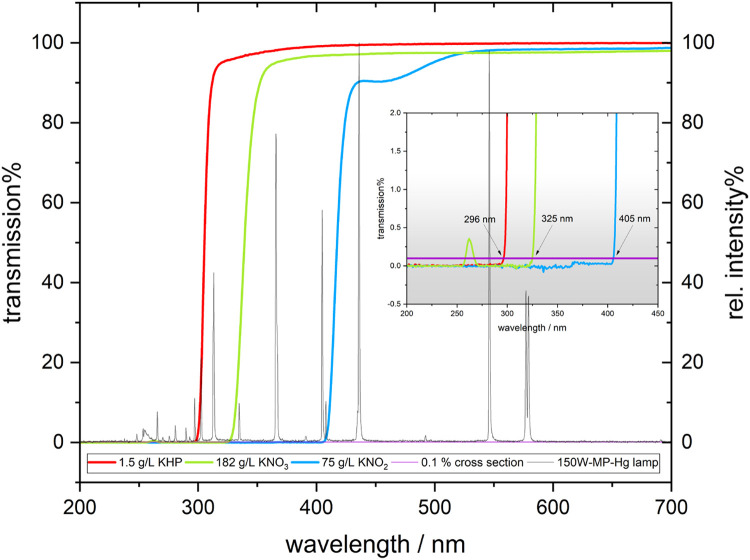
Transmission spectra of 1.5 g/L KHP, 182 g/L KNO_3_, 75 g/L KNO_2_ and 150 Watt-MP-Hg lamp spectra (grey). The inset shows the intersections of the 0.1 % cross section with the respective spectra of the cut-off filter.

To address this challenge, our experimental approach incorporates the use of cut-off filters. Potassium hydrogen phthalate (KHP), potassium nitrate (KNO_3_) and potassium nitrite (KNO_2_) were evaluated as filter solutions [9]. The MP-Hg lamp used provides broad UV–visible emission, as shown in Fig. 5. The three cut-off solutions selected function as selective filters for the different UV regions. Specifically:

• KNO_2_ transmits primarily visible light.
• KNO_3_ transmits visible light and UVA.
• KHP transmits visible light, UVA, and UVB.
• Without a filter, the full lamp spectrum, including UVC, is transmitted.

These filters enable controlled studies of photocatalytic and photolytic processes involving various micropollutants and semiconductors. Importantly, they also allow for simulating natural variations in the solar spectrum—particularly in the short-wavelength region, to assess its impact on photocatalysis. Consequently, our MAPMR system may facilitates the evaluation of materials for solar-driven photocatalysis under reproducible laboratory conditions. To prevent any interaction between the cut-off filters and the photocatalytic reaction, such as the potential formation of reactive nitrogen species from KNO_3_ and KNO_2_ [10], the filters were introduced into the cooling solution flowing through the void annular region of the annular quartz tube, rather than into the reaction solution [11]. This configuration ensures that the filters modify the light spectrum without affecting the reaction, while the cooling solution maintains the desired temperature.

Based on the absorption spectra of AMX and the reflectance spectra of TiO_2_-PES, KNO_3_ was selected as the UV cut-off filter solution with a 325 nm cut-off. This enables differentiation between photocatalytic and photolytic processes in AMX degradation by comparing results from experiments conducted with and without the KNO_3_ filter solution.

The photocatalytic degradation of AMX with TiO₂-PES in the MAPMR was conducted under semi-batch conditions. The reactor was initially filled with 0.285 L of ultrapure water containing 5 mM phosphate buffer, with the pH carefully adjusted to 7 using diluted solutions of NaOH or H_3_PO_4_. To ensure continuous flow, the flow rate was set at 1 L/min, and the aqueous solution within the reactor was magnetically stirred at 2000 rpm to maintain uniform mixing. Prior to the experiments, the MP-Hg lamp was turned on and allowed to pre-heat and stabilize for 20 min. After the MP-Hg lamp stabilized, the required volume of AMX stock solution (typically 100 mL of a 1.5 mM solution, pH 7) was added to the external reservoir, achieving a final AMX concentration of 50 µM (18 mg/L) throughout the reactor system. Following the addition of the stock solution, uniform distribution within the system was achieved in approximately 30 s, as confirmed in a separate experiment described in Section S8.

The degradation of AMX was examined through photolysis, adsorption onto the PES membrane and photocatalysis. Photolysis experiments were performed in the absence of the membrane under different light irradiation conditions, including without a cut-off filter solution, as well as with 61 g/L KNO₃ and with 182 g/L KNO₃. For the adsorption experiments, the TiO₂-PES was placed in the reactor in the dark. Both adsorption and photocatalytic experiments utilized all three reactor slots, each containing a 7.5 × 9.5 cm TiO₂-PES membrane sample (3.6 mg TiO₂/cm²) secured in a bracket holder (Fig. 2), resulting in a total effective membrane area of 214 cm².

**Fig. 6:**
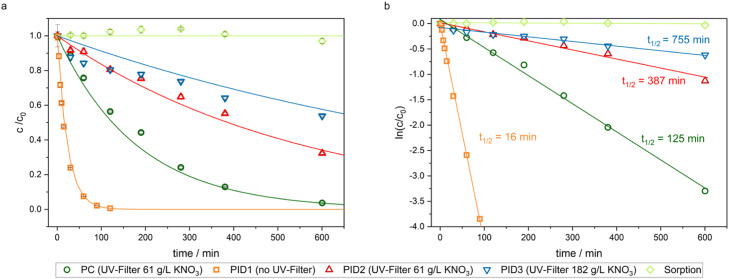
**a)** Removal of 50 µM AMX for different reaction conditions. **b)** Corresponding ln-linearised degradation curves. PC: Photocatalysis, PID: Photoinduced dissociation. t_1/2_: half-lives.

The process parameters, including pH, dissolved oxygen (DO), lamp spectrum, flow rate, and temperature, were monitored throughout the experimental period. These parameters remained stable during the investigation, as demonstrated by control charts for all experiments (see SI, Section S9). Only the temperature increased by approximately 5 °C at the beginning of the tests. Sampling was conducted over 10 h using an automated fraction collector, and the samples were analyzed using LC-ESI-HRMS (Orbitrap) for quantification of residual AMX (SI, Section S7). The degree of AMX degradation was determined by dividing the AMX concentration at a given reaction time by the initial concentration, based on the LC-ESI-HRMS peak areas (Fig. 6a). These data were further transformed using the natural logarithm to derive pseudo-first-order rate constants through linear regression analysis (Fig. 6b).

As shown in Fig. 6a), direct photolysis of AMX resulted in complete degradation within 100 min, as expected from the overlap of the absorption and emission spectra shown in Fig. 4. UV cut-off filters decreased the direct photolysis rate, with 60 % and 40 % degradation observed with 61 g/L and 182 g/L KNO_3_, respectively, over 600 min. Although KNO_3_ effectively blocks UV light below 325 nm (Fig. 4), the photodegradation of AMX above λ > 360 nm can be attributed to minimal light absorption by AMX extending into the visible spectrum above 400 nm (Fig. 5) [12,13]. The photolytic half-life of AMX increased from 387 min to 755 min as the KNO₃ concentration increased from 61 g/L to 182 g/L, further indicating a concentration-dependent suppression of light intensity below 325 nm (Fig. 6b). Overall, using nitrate as a UV-filter extended the half-life of AMX by up to 47-fold compared to photolysis without a UV-filter.

Subsequently, photocatalytic degradation experiments were conducted using the TiO₂-PES membrane with 61 g/L KNO₃ as a cut-off filter, yielding an AMX half-life of approximately 125 min (PC, dark green). This represents a significant reduction compared to the 387-min half-life observed in the photolysis experiment with the 61 g/L KNO₃ cut-off filter, suggesting that the difference can be entirely attributed to photocatalytic degradation. To further confirm this, control experiments conducted in the dark showed that the AMX concentration did not change for 600 min when in contact with the TiO₂-PES (Fig. 6a, light green), demonstrating that there was no contribution from adsorption towards the photocatalytic degradation of AMX. All kinetic parameters are summarized in Table 1.

**Table 1:** Pseudo first-order reaction rates, half-lives, and coefficient of determination of the degradation experiments.

		Photocatalysis (61 g/L KNO_3_)	PID1 (no UV-Filter)	PID2 (61 g/L KNO_3_)	PID3 (182 g/L KNO_3_)
k	1/min	5.53E-03	0.04	1.79E-03	9.18E-04
t _1/2_	min	125	16	387	755
Coefficient of determination (R^2^)		0.996	0.999	0.982	0.959

To ensure the reusability of the TiO_2_-PES membrane, additional experiments were conducted, confirming its potential for multiple uses in compound degradation. The corresponding data is provided in the SI (SI, section S5).

## Conclusion

This study presents the ‘Modular Annular Photocatalytic Membrane Reactor (MAPMR)’ designed to investigate the mechanisms of photocatalytic processes in aqueous solutions. Key process parameters can be thoroughly monitored during photocatalytic degradation experiments, ensuring standardized conditions for reliable comparison across test runs. Such detailed monitoring is often neglected in photocatalyst research, hindering systematic material comparisons. Using chemical wavelength cut-off filters further differentiated photolysis and photocatalysis contributions. The system's effectiveness was demonstrated through the photocatalytic degradation of amoxicillin (AMX) using porous TiO₂-immobilized polymer membranes. Validation with AMX as a model contaminant highlights the reactor's ability to enhance degradation efficiency and facilitate detailed kinetic analyses. These findings underscore the MAPMR's potential to advance sustainable and efficient photocatalytic reactor designs, offering a promising approach for addressing organic pollutants in water treatment.

## Limitations

None.

## Ethics statements

None.

## CRediT author statement

**Michael S. Leupold**: Conceptualization, Methodology, Investigation, Visualization, Software, Writing – original draft, Writing – review & editing. **Max Reuschenbach:** Conceptualization. **Gerrit Renner:** Data curation – review & editing. **Klaus Kerpen:** Conceptualization – review & editing. **Anam Asghar:** Writing – review & editing. **Lukas Fischer**: Writing – review & editing. **Torsten C. Schmidt:** Funding acquisition– review & editing.

## Declaration of competing interest

The authors declare that they have no known competing financial interests or personal relationships that could have appeared to influence the work reported in this paper.

## Data Availability

Data will be made available on request.

## Appendix Supplementary materials

Image, application 1
