# Supplementary material for: Modular annular photocatalytic membrane reactor for the degradation of micropollutants: Design and application
Source: MethodsX. 2025 Jul 1;15:103478. doi: 10.1016/j.mex.2025.103478 (PMC12343478; doi:10.1016/j.mex.2025.103478)
Supplement: Supplementary file 1 [file mmc1.docx]

**Modular Annular Photocatalytic Membrane Reactor for Degradation of Micropollutants: Design and Application**

Michael S. Leupold^a, b^, Max Reuschenbach^a, b^, Gerrit Renner^a, b^, Anam Asghar^a, b^, Klaus Kerpen^a^, Lukas Fischer^b^, Torsten C. Schmidt^a, b, d^

*^a^Instrumental Analytical Chemistry, University of Duisburg-Essen, Universitätsstr.5, Essen 45141, Germany.*

*^b^Centre for Water and Environmental Research (ZWU), University of Duisburg-Essen, Universitätsstr.2, Essen 45141, Germany.*

*^c^Technical Chemistry II, University of Duisburg-Essen, Universitätsstr.5, Essen 45141, Germany.*

*^d^IWW Water Center, Moritzstr.26, Mülheim an der Ruhr 45476, Germany.*

Supplementary data

**Corresponding author**

| **Subject area** | Chemistry |
| --- | --- |

Anam Asghar

Email: [anam.asghar@uni-due.de](mailto:anam.asghar@uni-due.de)

Tel. +49 201 183-6778

Fax. +49 201 183-6773

**Supplementary information**

## **S1 Chemicals**

Amoxicillin (AMX; purity 95.0-102.0%), potassium nitrate (KNO_3_ analytical grade), potassium nitrite (KNO_2,_ analytical grade), barium sulfate (BaSO_4_, analytical grade), titanium(IV) oxide(TiO_2_, a mixture of rutile and anatase, nano powder, <100 nm particle size), H_2_O (LC-MS grade), acetonitrile (ACN, LC-MS grade), formic acid (FA, analytical grade), phosphoric acid (H_3_PO_4_, analytical grade), sodium hydroxide (NaOH), sodium phosphate (Na_3_PO_4_, analytical grade), sodium hydrogen phosphate (Na_2_HPO_4_, analytical grade), Potassium hydrogen phthalate (KHP), potassium indigo trisulphonate were purchased from Sigma-Aldrich, City, Germany. AMX stock solutions were always freshly prepared one day before use and stored at 4°C until use.

## **S2 Scanning electron microscopy (SEM) and energy-dispersive X-ray spectroscopy (EDX)**

For the SEM and EDX analyses, the TiO_2_-PES membrane was frozen in liquid nitrogen and fractured to expose the cross-section. The sample was sputter-coated with PdAu, and analyzed using an Apreo S LoVac system (Thermo Fisher Scientific). SEM images were acquired under a pressure of 10^-3^ Pa, with an accelerating voltage of 5 kV and a beam current of 13 pA. For EDX analysis, an accelerating voltage of 30 kV was applied.

## **S3 Band gap determination**

The UV 2600-i Shimadzu UV-visible spectrophotometer (Shimadzu), coupled with the ISR-2600 integrating sphere attachment (Shimadzu), was used to measure reflectance spectra and determine the band gap. Each measurement was conducted with a slit width of 2 nm, a medium scan speed, and a data interval of 1. The measurements were carried out using the LabSolutions UV-Vis software (Shimadzu). Before each experiment, the system was baselined with pure BaSO_4_ over the wavelength range of 200 to 800 nm. TiO_2_ powder was then added to the sample holder to ensure complete coverage. The reflectance spectra of the sample were subsequently recorded.

The same procedure was applied to a round piece of the TiO_2_ polyether sulfone membrane (TiO_2_-PES) and a PES membrane. Both membrane samples were fully inserted into the sample holder. Additionally, a membrane exposed to AMX during a degradation experiment was analyzed to assess material changes. The membrane turned yellow after exposure, consistent with the yellow coloration of the reaction solution caused by AMX. Conversely, irradiation without AMX caused no staining, providing visual evidence of transformation products binding to the membrane, likely diminishing the TiO_2_-PES performance.

The band gaps of the samples were determined using the Band Gap Calculating Excel Macro (Shimadzu), assuming indirect allowed transitions (n = 2) and employing the Tauc method [1, 2]. The PES membrane's band gap was determined using a direct allowed transition (n = 1/2). The absorption coefficient was derived from the reflectance data (R%) using the following equation (Eq. 1):

| $\alpha=\frac{\left( 1-\frac{R}{100} \right)^{2}}{\frac{2R}{100}}$ | [Eq.1] |
| --- | --- |

Figure S1 (a) presents the reflectance spectra of the respective specimen, while Figure S1(b) displays the corresponding Tauc plots. The calculated bandgaps are summarized in Table S1.





***Figure S1 | (a)*** *Reflectance spectra of TiO_2_ and support material,* ***(b)*** *Tauc plots of the reflectance spectra*

***Table S1 |*** *Linear equation determined by the Tauc Method*

| Sample | Transition | Bandgap [eV] | Slope [1/eV] | y-axis |
| --- | --- | --- | --- | --- |
| TiO_2_ powder | 2 | 3.20 | 16.95 | -54.54 |
| TiO_2_ PES | 2 | 3.12 | 12.53 | -39.09 |
| TiO_2_ PES after use | 2 | 2.93, 3.5 | 7.43 | -21.78 |
| PES | 0.50 | 4.01 | 3444.10 | -13801 |

## **S4 Determination of the contact angle of the TiO_2_-PES membrane**

The membrane was first dried, and then the water contact angle was measured using the OCA15 system from Dataphysics. A 5 μL droplet of deionized water was placed on the top side and then the bottom sides of the membrane, and the contact angles were recorded. Corresponding images are provided in Figure S2.


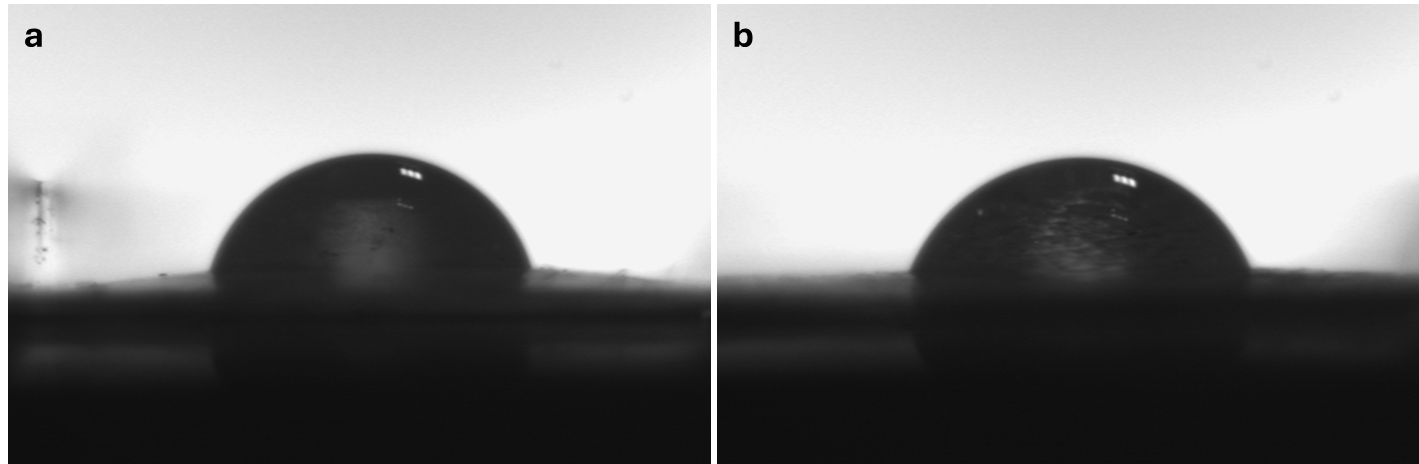


**Figure S2** | Images to determine the contact angle of the TiO_2_-PES membranes a) top side b) underside

The water contact angle was determined to be 76.0° on the top side and 71.4° on the bottom side of the membrane.

## **S5 Membrane reuse experiments**

To evaluate the reusability of the TiO_2_-PES membrane, a 16 cm^2^ piece of the membrane was tested under standard degradation conditions used in this study. Specifically, 100 mL of a 50 µM AMX solution in 5 mM phosphate buffer (pH7) prepared with ultra-pure water was used. The experiment was conducted five times using the same membrane piece in a similar, smaller reactor setup. The total irradiation time was 25 h. The results are shown in Figure S3.





**Figure S3 |** Reusability of the TiO2-PES membrane.

The data showed no noticeable change in membrane performance over the five cycles, confirming the reusability.

## **S6 NPOC measurements**

The organic carbon content during degradation was determined using a total organic carbon (TOC) analyzer, TOC-L (Shimadzu). Organic carbon was measured as non-purgeable organic carbon (NPOC). The analyzer parameters are provided in Table S2. KHP dissolved in ultrapure water was used as the TOC standard. The stock solution of the TOC standard, prepared at a concentration of 1000 mg_C_/L, was stored at 7°C and assumed to remain stable for two weeks. The setting used for NPOC analysis are provided in Table S2

***Table S2 |*** *TOC-L settings*

| Parameter | Setting |
| --- | --- |
| Analysis | NPOC |
| Number of injections | 3/5 |
| Number of washes | 2 |
| SD Max | 0.1 |
| CV Max | 2% |
| Sparge gas flow | 80 mL/min |
| Sparge time | 2:00 min |
| Acid addition | 10% |
| Injection volume | 50 µL |
| Calibration | 0, 5, 10, 20,30, 40, 50 mg/L |





***Figure S4 |*** *NPOC-data on three amoxicillin degradation experiments. ([AMX]_i_ = 50 µM, 5 mM phosphate, pH7, UV-Filter 61g/L KNO_3_, 1.5 g/L KHP). PC: Photocatalysis, PID: Photoinduced dissociation*

## **S7 LC-HRMS**

Separation of AMX was carried out using a DIONEX UltiMate 3000 Autosampler and a DIONEX UltiMate 3000 Pump, equipped with a Luna Omega Polar C18 column (100 Å pore size, 5 µm particle size,100 x 2.1 mm). The mobile phase consisted of a gradient of H_2_O and ACN, both acidified with 0.1% (v/v) formic acid, as detailed in Table S3. The injection volume was 10 µL. The column oven was maintained at 25.0±1.0 °C, and the autosampler rack was held at a temperature of 15°C.

***Table S3 |*** *LC-gradient used to separate amoxicillin and its transformation products. A: 0.1% formic acid (v/v) H_2_O, B: 0.1% formic acid (v/v) in acetonitrile, Curve: 5 = linear line.*

| Time (min) | Flow (ml/min) | %B | Curve |
| --- | --- | --- | --- |
| 0 | 0.4 | 0 | 5 |
| 1 | 0.4 | 0 | 5 |
| 2 | 0.4 | 3 | 5 |
| 9 | 0.4 | 10 | 5 |
| 10 | 0.4 | 20 | 5 |
| 14 | 0.4 | 90 | 5 |
| 15 | 0.4 | 90 | 5 |
| 16 | 0.4 | 0 | 5 |
| 21 | 0.4 | 0 | 5 |

***Table S4 |*** *Q executive™ Orbitrap Settings*

| Full MS |  |
| --- | --- |
| Resolution | 70 000 |
| AGC target | 1e6 |
| Maximum IT | 100 ms |
| Scan range | 80 to 800 m/z |
| dd-MS2 | 17 500 |
| AGC | 5e4 |
| Maximum IT | 50 ms |
| Loop count | 5 |
| TopN | 5 |
| Isolation window | 1.4 m/z |
| CE | 30 |
| Dd settings |  |
| Minimum AGC target | 8.00e2 |
| Intensity threshold | 1.6e4 |
| Dynamic exclusion | 3.0 s |
| Global settings |  |
| User role standard |  |
| Use lock masses | off |
| Chrom peak width (FWHM) | 6 s |
| Method duration | 21.00 min |
| HESI source |  |
| Run | 0-21 min |
| Polarity positive |  |
| Default charge state 1 |  |
| Sheath gas flow rate | 50 |
| Aux ga flow rate | 15 |
| Sweep gas flow rate | 2 |
| Spray voltage | 3.5 kV |
| Capillary temp | 350 °C |
| S-lens RF level | 50.0 |
| Aux gas heater temp | 150 °C |

## **S8 Mixing experiments**

The experimental setup was designed to allow the spiking of probe compounds and sampling during degradation experiments at the external reservoir to ensure safe system operation, thereby preventing direct contact with the light source. Accordingly, it was necessary to determine the time required for the probe compound to be introduced into the external reservoir to achieve homogeneous mixing within the system while the pump and stirrer were operating.

The setup was modified to include a flow-through cuvette, a USB spectrometer (OceanOptics 2000, Ocean Optics), and a peristaltic pump set at 990 rpm to evaluate the mixing efficiency of the reactor system. These parts were not included in regular photocatalytic experiments. These experiments were conducted without a light source, with only the pump and stirrer in operation.


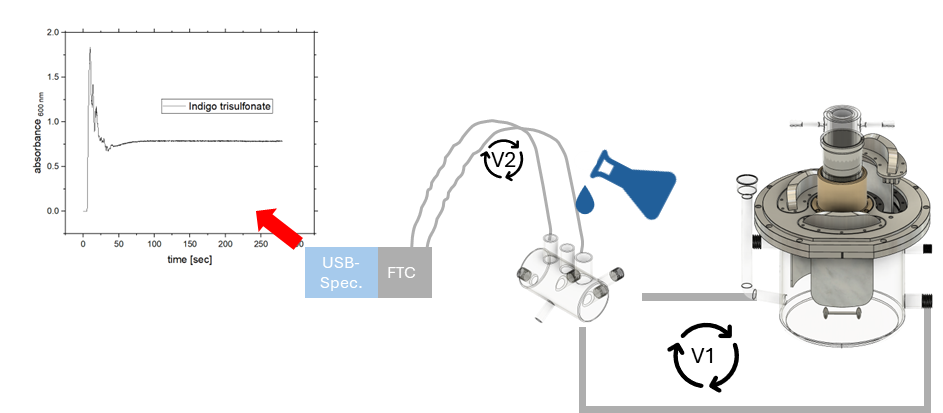


***Figure S5 |*** *Experimental Setup to test mixing properties of the Photocatalytic-Reactor with a 50 µM indigo trisulfonate solution. FTC (Flow-through cuvette). The discharged volume for the flow-through cuvette is negligibly small compared to the reactor volume (V1>>V2)*

A bypass system was established by feeding a small sample volume from the external reservoir into a flow-through cuvette using a peristaltic pump to continuously measure the absorbance of the solution, with the solution then returned to the external reservoir. The system was filled with 2900 mL of ultrapure water. The reactor stirrer was operated at 2000 rpm, and the pump flow rate was set at 1.0 L/min, 1.5 L/min and 2.0 L/min in three different experiments.

For testing, 100 mL of 1.5 mM potassium indigo trisulphonate solution was introduced into the external reservoir, resulting in a final concentration of 50 µM after homogeneous mixing in the system. Absorbance at 600 nm was monitored over time using the USB spectrometer connected to the flow cell. Homogeneous mixing was confirmed when the absorbance at 600 nm remained stable. Figure S 4A illustrates the absorbance as a function of time.

As shown in Figure S 6, initially spiking the indigo stock solution results in a high concentration in the external reservoir, increasing absorbance within the first 20-30 s, depending on the flow rate. This is attributed to the locally elevated concentration during the reservoir filling. Due to continuous flow, the flow-through cuvette draws the concentrated solution from the external reservoir, causing the indigo concentration to be high during the initial seconds post-injection. The concentrated solution is subsequently transferred from the external reservoir to the reactor, leading to a decrease in concentration until a stable absorbance is achieved.

To better evaluate the mixing behavior, the absorbance data were processed using the Savitzky-Golay filter (2nd order polynomial, 10 points of the windows), followed by differentiation concerning time, squaring, and reintegrating concerning time, and normalization to a range of 0-100 %. A boundary condition was applied, requiring the processed data to exceed a threshold of 99 % to confirm homogeneous mixing in the system. The processed absorbance data is presented in Figure S6 (b).


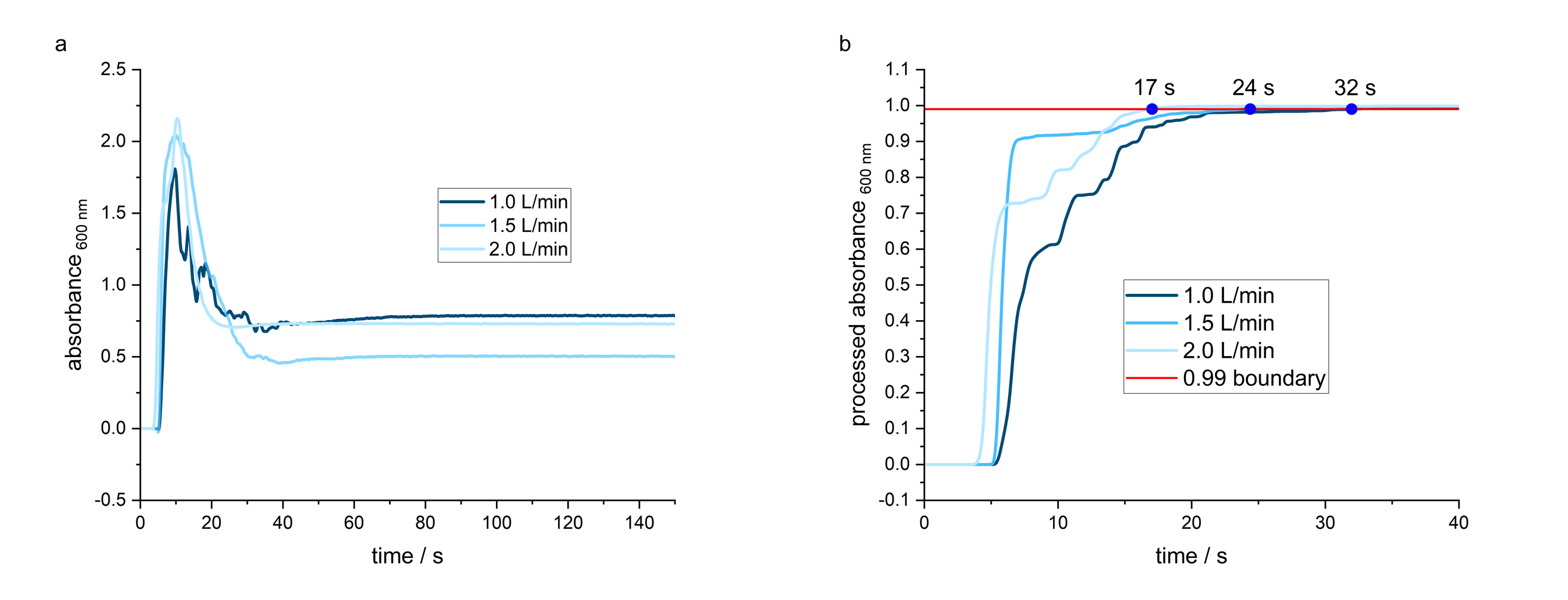


***Figure S6 |*** *Mixing experiment in the reactor system. (a) determined absorbance over time after indigo spiking (b) processed absorbance data*

The points where the processed absorbance curves intersect the 99 % threshold indicate when the system reached homogeneity. Varying the pump flow rate from 1.0 to 2.0 L/min yielded expected trends, with shorter mixing times observed at higher flow rates. However, the mixing time at the lowest test flow rate was 32 s, corresponding to the minimum sampling interval. Since the degradation process occurs more slowly than the mixing process, a flow rate of 1.0 L/min was selected to prevent leakage and reduce excessive pressure on the glass apparatus.

## **S9 On-line data**

Figure S7 presents exemplary measurement data from a degradation experiment. The monitoring confirmed that the reaction conditions were reproducible throughout 10 h.


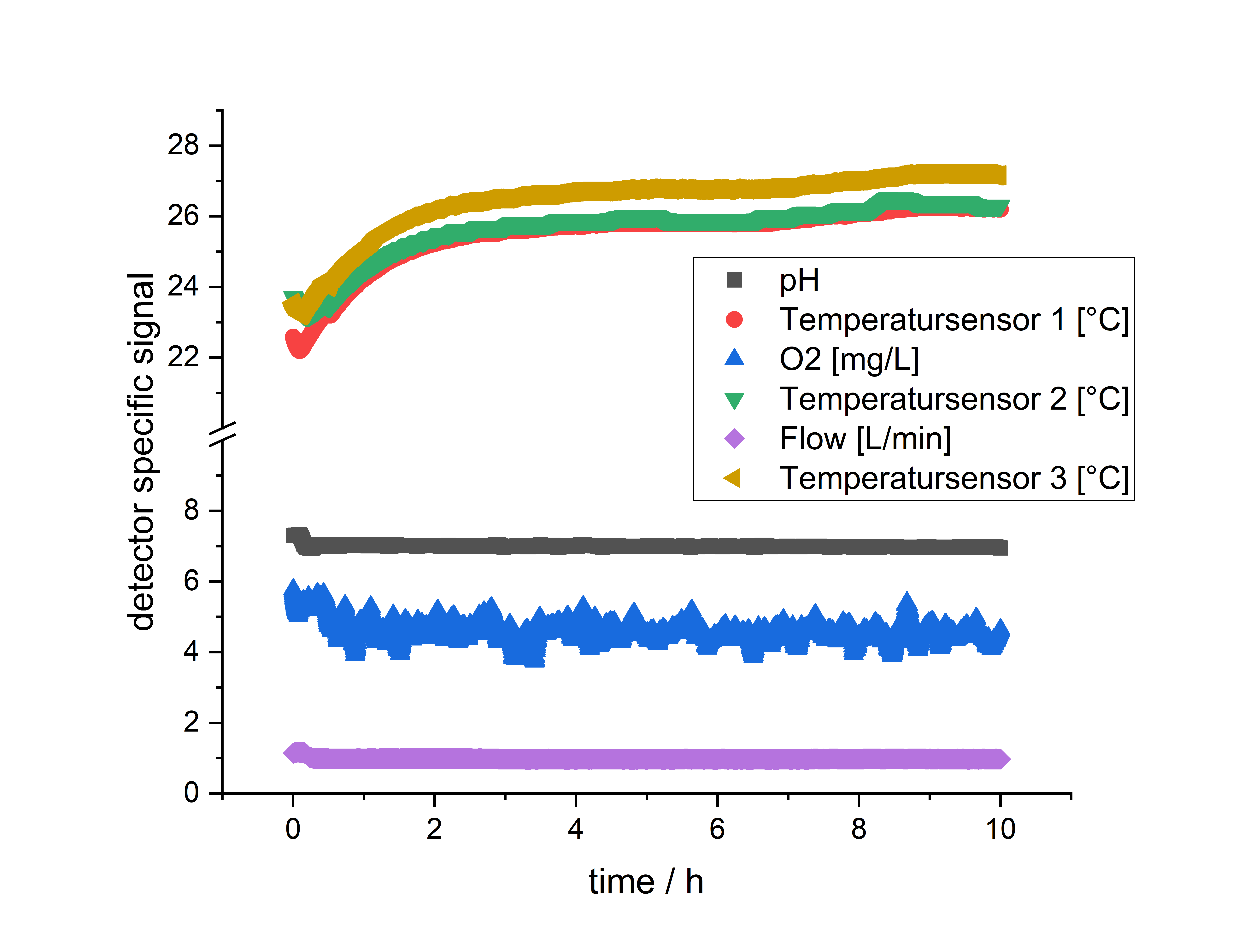


***Figure S7 |*** *Exemplary online data were merged via a LabVIEW code*

## **S10 Pictures of the experimental Setup and the reactor**


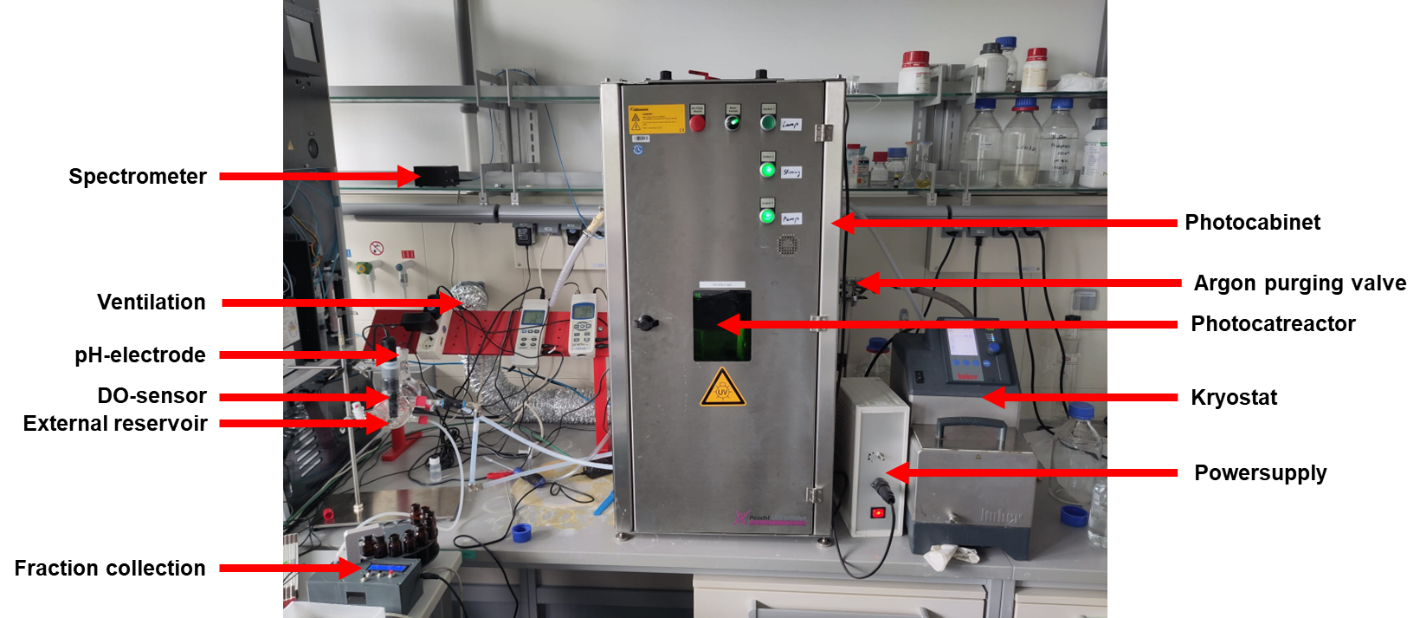


***Figure S8 |*** *Experimental setup used in this study*


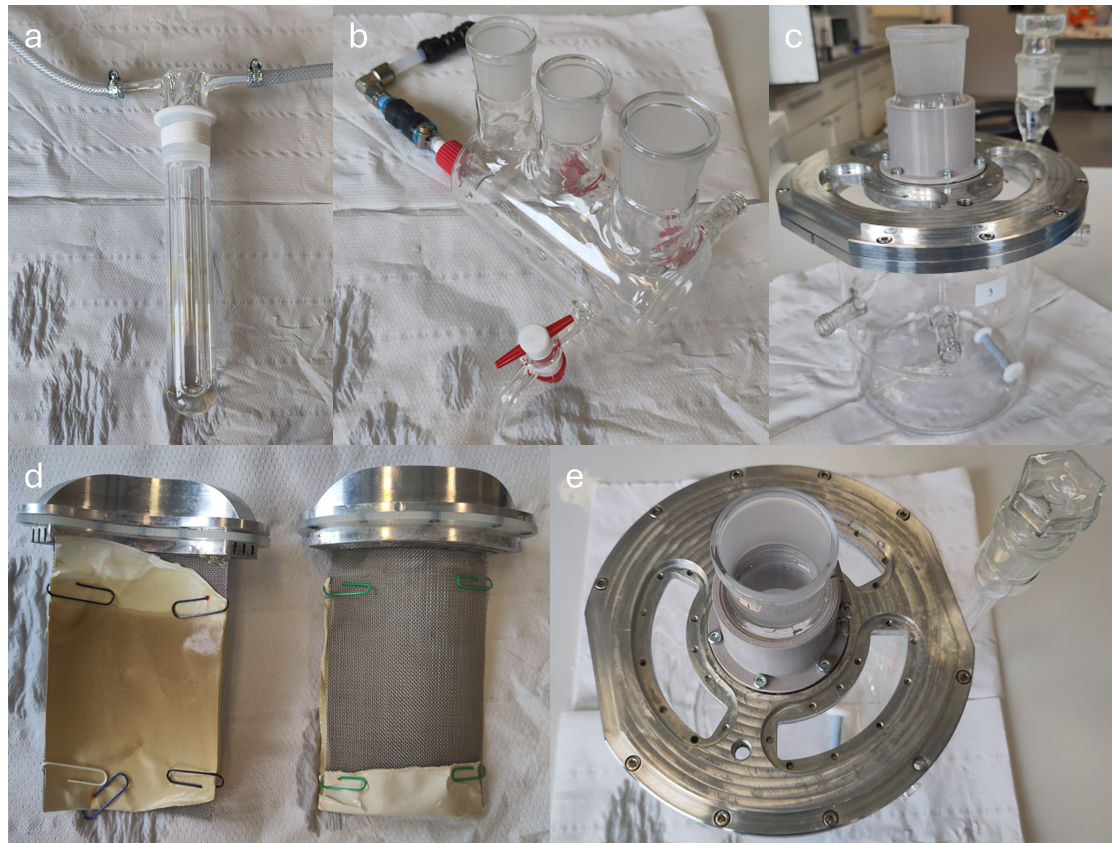


***Figure S9 |*** *MAPMR parts. a) cooling unit, b) external reservoir (outer vessel), c) reactor chamber, d) Catalyst bracket and used TiO_2_-PES membrane, e) reactor flange.*

## **S11 Blueprints**

**References**

[1] J. Tauc, Optical properties and electronic structure of amorphous Ge and Si, Materials Research Bulletin, 3 (1968) 37-46.

[2] P. Makuła, M. Pacia, W. Macyk, How To Correctly Determine the Band Gap Energy of Modified Semiconductor Photocatalysts Based on UV-Vis Spectra, Journal of Physical Chemistry Letters, 9 (2018) 6814-6817.
